# *Lotus japonicus* Symbiosis Genes Impact Microbial Interactions between Symbionts and Multikingdom Commensal Communities

**DOI:** 10.1128/mBio.01833-19

**Published:** 2019-10-08

**Authors:** Thorsten Thiergart, Rafal Zgadzaj, Zoltán Bozsóki, Ruben Garrido-Oter, Simona Radutoiu, Paul Schulze-Lefert

**Affiliations:** a Max Planck Institute for Plant Breeding Research, Cologne, Germany; b Cluster of Excellence on Plant Sciences, Max Planck Institute for Plant Breeding Research, Cologne, Germany; c Department of Molecular Biology and Genetics, Faculty of Science and Technology, Aarhus University, Aarhus, Denmark; Mass General Hospital

**Keywords:** microbiome, plant-microbe interactions, symbiosis

## Abstract

The wild legume Lotus japonicus engages in mutualistic symbiotic relationships with arbuscular mycorrhiza (AM) fungi and nitrogen-fixing rhizobia. Using plants grown in natural soil and community profiling of bacterial 16S rRNA genes and fungal internal transcribed spacers (ITSs), we examined the role of the *Lotus* symbiosis genes *RAM1*, *NFR5*, *SYMRK*, and *CCaMK* in structuring bacterial and fungal root-associated communities. We found host genotype-dependent community shifts in the root and rhizosphere compartments that were mainly confined to bacteria in *nfr5* or fungi in *ram1* mutants, while *symrk* and *ccamk* plants displayed major changes across both microbial kingdoms. We observed in all AM mutant roots an almost complete depletion of a large number of Glomeromycota taxa that was accompanied by a concomitant enrichment of Helotiales and Nectriaceae fungi, suggesting compensatory niche replacement within the fungal community. A subset of Glomeromycota whose colonization is strictly dependent on the common symbiosis pathway was retained in *ram1* mutants, indicating that *RAM1* is dispensable for intraradical colonization by some Glomeromycota fungi. However, intraradical colonization by bacteria belonging to the *Burkholderiaceae* and *Anaeroplasmataceae* is dependent on AM root infection, revealing a microbial interkingdom interaction. Despite the overall robustness of the bacterial root microbiota against major changes in the composition of root-associated fungal assemblages, bacterial and fungal cooccurrence network analysis demonstrates that simultaneous disruption of AM and rhizobium symbiosis increases the connectivity among taxa of the bacterial root microbiota. Our findings imply a broad role for *Lotus* symbiosis genes in structuring the root microbiota and identify unexpected microbial interkingdom interactions between root symbionts and commensal communities.

## Abstract (precis)

Studies on symbiosis genes in plants typically focus on binary interactions between roots and soilborne nitrogen-fixing rhizobia or mycorrhizal fungi in laboratory environments. We utilized wild type and symbiosis mutants of a model legume, grown in natural soil, in which bacterial, fungal, or both symbioses are impaired to examine potential interactions between the symbionts and commensal microorganisms of the root microbiota when grown in natural soil. This revealed microbial interkingdom interactions between the root symbionts and fungal as well as bacterial commensal communities. Nevertheless, the bacterial root microbiota remains largely robust when fungal symbiosis is impaired. Our work implies a broad role for host symbiosis genes in structuring the root microbiota of legumes.

## INTRODUCTION

Mutualistic plant-microbe interactions are essential adaptive responses dating back to plant colonization of terrestrial habitats (1, 2). Endosymbiotic association with obligate arbuscular mycorrhizal (AM) fungi belonging to the phylum Glomeromycota is considered to have enabled early land plants to adapt to and survive under harsh edaphic conditions by improving the acquisition of nutrients, especially phosphorus, from soil (3). It is estimated that approximately 80% of extant plant species remain proficient in AM symbiosis (AMS), testifying to its importance for survival in natural ecosystems (4–6). Another more recent endosymbiotic relationship has evolved between plants belonging to distinct lineages of flowering plants (Fabales, Fagales, Cucurbitales, and Rosales) and nitrogen-fixing members of the *Burkholderiales*, *Rhizobiales*, or *Actinomycetales*, enabling survival on nitrogen-poor soils. These bacteria fix atmospheric nitrogen under the low-oxygen conditions that are provided by plant root nodules (RNs).

Studies using mutant legumes deficient in both AMS and root nodule symbiosis (RNS) revealed that a set of genes defined as the common symbiotic signaling pathway (CSSP) are crucial for these symbioses. In the model legume Lotus japonicus, Nod factor perception by NFR1 and NFR5 activates downstream signaling through SYMRK, a malectin- and leucine-rich repeat (LRR)-containing receptor-like kinase (RLK) (7), currently considered to be the first component of the CSSP. SYMRK associates with NFR5 through a mechanism involving intramolecular cleavage of the SYMRK ectodomain, thereby exposing its LRR domains (8). Signaling from the plasma membrane is transduced to the nuclear envelope, where ion channels (9, 10), nuclear pore proteins (11–13), and cyclic nucleotide-gated channels (14) mediate symbiotic calcium oscillations. These calcium oscillations are interpreted by the calcium- and calmodulin-dependent protein kinase CCaMK (15, 16), which interacts with the DNA binding transcriptional activator CYCLOPS (17–19). Several GRAS transcription factors (NSP1, NSP2, RAM1, and RAD1) are activated downstream of CCaMK and CYCLOPS and determine whether plants engage in AMS or RNS.

Plants establish symbioses with AM fungi and nitrogen-fixing bacteria by selecting interacting partners from the taxonomically diverse soil biome. These interactions are driven by low mineral nutrient availability in soil and induce major changes in host and microbial symbiont metabolism (20, 21). Although RNS develops as localized events on legume roots, analysis of *Lotus* mutants impaired in their ability to engage in symbiosis with nitrogen-fixing bacteria revealed that these mutations not only abrogate RNS but also impact the composition of taxonomically diverse root- and rhizosphere-associated bacterial communities, indicating an effect on multiple bacterial taxa that actively associate with the legume host, irrespective of their symbiotic capacity (22). In contrast, the effect of AMS is known to extend outside the host via a hyphal network that can penetrate the surrounding soil and even indirectly affect adjacent plants (23). In soil, fungal hyphae themselves represent environmental niches and are populated by a specific set of microbes (24). Although the biology of AM fungi is well understood, and genetic disruption of AMS was recently shown to exert a relatively small effect on root-associated fungal communities in *Lotus* (25), the potential impact of AMS and/or RNS on root-associated bacterial and fungal commensals remains poorly understood, mainly because previous studies have focused on either bacteria (22) or fungi (25) alone.

We reasoned that the model legume *Lotus japonicus*, with its well-characterized symbiosis signaling mutants impaired in RNS, AMS, or both, is particularly useful to examine whether genetic perturbations of these symbioses impact only commensal communities of the corresponding microbial kingdom and/or influence microbial interkingdom interactions in the root microbiota. We applied bacterial and fungal community profiling experiments to root samples collected from wild-type (WT) L. japonicus and four symbiosis signaling mutants, grown in natural soil. We show that genetic disruption of the symbioses results in significant host genotype-dependent microbial community shifts in the root and surrounding rhizosphere compartments. These changes were mainly confined to either bacterial or fungal communities in RNS- or AMS-deficient plant lines, respectively, whereas mutants with defects in the CSPP revealed major changes in assemblages of the root microbiota across both microbial kingdoms. We found that perturbation of AM symbiosis alone is sufficient to deplete a subset of bacterial taxa belonging to the *Burkholderiaceae* and *Anaeroplasmataceae* families from the root microbial community, whereas simultaneous perturbation of AM and *Rhizobium* symbioses increases the connectivity within the bacterial root cooccurrence network.

## RESULTS

### The root fractionation protocol affects the composition of associated bacterial communities.

**FIG 1:**
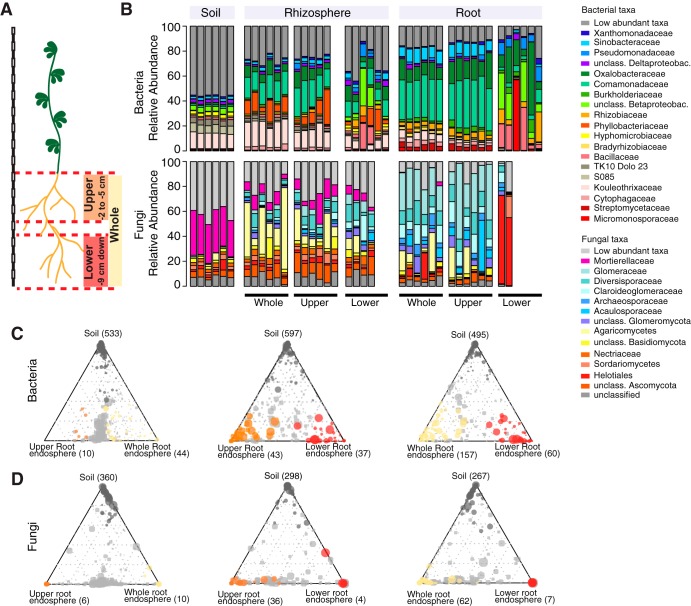
Bacterial and fungal community profiles for different root fractions of *L. japonicus*. (A) Cartoon showing the length of the three different root fractions. (B) Community profile showing the relative abundances of bacterial (top) and fungal (bottom) families across compartments and fractions (only samples with >5,000 reads [bacteria] or >1,000 reads [fungi] are shown, and taxa having an average RA of <0.1 [bacteria] or <0.15 [fungi] across all samples are aggregated as low-abundance taxa). (C) Ternary plots showing bacterial OTUs that are enriched in the endosphere of specific root fractions, compared to the soil samples. (D) Ternary plots showing fungal OTUs that are enriched in the endosphere of specific root fractions, compared to the soil samples. The circle size corresponds to the RA across all fractions. Dark-gray circles denote OTUs that are enriched in soil, and light-gray circles always represent OTUs that are not enriched in any of the fractions.

Previous physiological studies have shown that only cells of a specific developmental stage, located in the root elongation zone, respond to Myc and Nod factors, mount symbiotic calcium oscillations, and enable epidermal infection by rhizosphere-derived fungal and bacterial symbionts (26, 27). To explore the spatial organization of root-associated bacterial and fungal communities along the longitudinal axis, we collected samples of the upper and lower root zones as well as the entire root system of 10-week-old Gifu wild-type plants, grown in Cologne soil (2 to 5 cm and >9 cm of the root system, respectively) (Fig. 1A) (28). Microbial assemblages of these three root endosphere compartments were compared with the communities in the corresponding rhizosphere fractions, i.e., soil tightly adhering to the respective root zones, and with the bacterial biome present in unplanted Cologne soil. 16S rRNA gene amplicon libraries of the V5-V7 hypervariable region and gene libraries of the internally transcribed spacer 2 (ITS2) region of the eukaryotic ribosome were generated by amplification (29–31). Information on the number and relative abundance (RA) of operational taxonomic units (OTUs) in each compartment was used to calculate α-diversity (Shannon index; within-sample diversity), β-diversity (Bray-Curtis distances; between-sample diversity), OTU enrichment, and taxonomic composition. For bacteria, we observed a gradual decrease in α-diversity from unplanted soil to the rhizosphere and to the root endosphere compartments, a trend that was similar for each longitudinal root fraction. This suggests that winnowing of root commensals from the highly complex soil biome occurs in all tested root zones (see Fig. S1A in the supplemental material). Similar overall results were obtained for the fungal data set (Fig. S1B), but the decrease in diversity from unplanted soil toward the rhizosphere was mild or even lacking. The latter finding is similar to that of a recent study of root-associated fungi in nonmycorrhizal Arabidopsis thaliana plants sampled at three natural sites (32). Analyses of taxonomic composition and β-diversity revealed striking differences in the endosphere and rhizosphere compartments associated with the upper and lower root longitudinal fractions (Fig. S1C and D). The composition of bacterial and fungal taxa of the whole root closely resembled that of the upper root fraction (Fig. 1B), with only low numbers of OTUs being differentially abundant between these two compartments (Fig. 1C and D). This suggests that microbes colonizing the lower root fraction constitute only a small fraction of the entire *Lotus* root microbiota. Additionally, we observed higher sample-to-sample variation in the taxonomic profiles of the lower root zone compared to the upper root fractions and whole roots (Fig. 1B). This greater community variation in the developmentally younger region of *L. japonicus* roots might reflect a nascent root microbiota or greater variation in root tissue and adherent rhizosphere samples that we recovered from this root zone by our fractionation protocol. Based on the finding that whole-root and upper root compartments host comparable bacterial communities and given their greater stability, we decided to use the former for further analyses.

10.1128/mBio.01833-19.1 FIG S1 Alpha and beta diversity across root fractions. (A) Shannon diversity indices for 16S amplicon data for soil (*n* = 6); lower (*n* = 6), upper (*n* = 6), and whole (*n* = 6) root fractions; and the respective rhizosphere samples (*n* = 6 each). (B) Shannon diversity index for ITS2 amplicon data for soil (*n* = 6); lower (*n* = 2), upper (*n* = 6), and whole (*n* = 6) root fractions; and the respective rhizosphere samples (*n* = 6 each, except for the lower root fraction [*n* = 4]) (*P* < 0.05 by ANOVA with Tukey’s *post hoc* test). (C) Principal-coordinate analysis of Bray-Curtis distances for bacterial data. (D) Principal-coordinate analysis of Bray-Curtis distances for fungal data. Download FIG S1, EPS file, 1.3 MB. Copyright © 2019 Thiergart et al. 2019 Thiergart et al. This content is distributed under the terms of the Creative Commons Attribution 4.0 International license.

### Host genes needed for symbioses determine bacterial and fungal community composition of *L. japonicus* root and rhizosphere.

**FIG 2:**
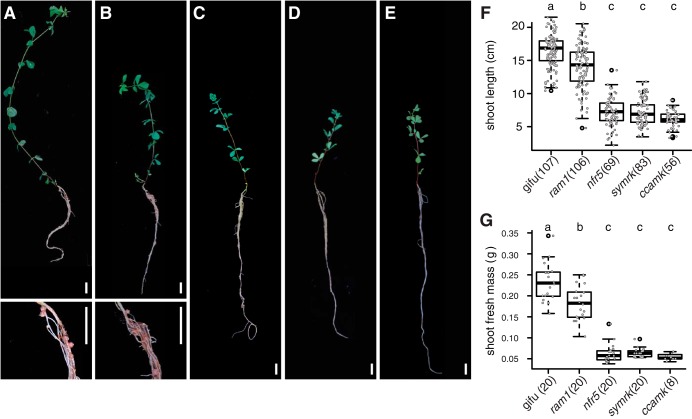
Phenotypes of WT and mutant plants. (A to E) Images depicting *L. japonicus* wild type (A) and *ram1* AMS-deficient (B), *nfr5* RNS-deficient (C), *symrk* AMS- and RNS-deficient (D), and *ccamk* AMS- and RNS-deficient (E) mutant plants. Insets show closeup views of nodules. Bars, 1 cm. (F) Box plots displaying the shoot length for the same set of genotypes as the one presented panels A to E. (G) Box plots displaying the shoot fresh mass. Letters above plots correspond to groups based on Tukey’s HSD test (*P* < 0.05). Numbers of samples are indicated in parentheses.

For root microbiota analysis, we cultivated WT (ecotype Gifu) *L. japonicus* and *nfr5-2*, *symrk-3*, *ccamk-13*, and *ram1-2* (*nfr5*, *symrk*, *ccamk*, and *ram1*, from henceforth) mutants in parallel in two batches of Cologne soil, to account for batch-to-batch and seasonal variation at the sampling site. *nfr5-2* mutant plants are impaired in rhizobial Nod factor perception and signaling, which prevents initiation of infection thread formation (33). Mutations in *SymRK* and *CCaMK* affect the common symbiosis pathway downstream of Nod or Myc factor perception, abrogating infection by either nitrogen-fixing rhizobia or AM fungi (7, 34). The RAM1 transcription factor controls arbuscule formation, and while *ram1* mutants of *L. japonicus* are indistinguishable from WT and permit incipient AM fungus infection, fungal colonization is terminated with the formation of stunted symbiotic structures (35). All plant genotypes appeared healthy (Fig. 2A to E), but the shoot length and shoot fresh weight of all mutant plants were significantly reduced in comparison to the WT (Fig. 2F and G), suggesting that genetic disruption of either AM or *Rhizobium* symbiosis is detrimental for the fitness of plants grown in natural soil. All genetic defects in nitrogen-fixing symbiosis, validated by the absence of root nodules in plants of the *nfr5*, *symrk*, and *ccamk* genotypes (Fig. 2C to E and Table S1), resulted in similarly severe impacts on plant growth (Fig. 2F and G), whereas both shoot length and shoot fresh weight were significantly but less severely reduced in *ram1* plants. *ram1* plants still formed nodules and, unlike WT and *nfr5* plants, showed impairment in AM symbiosis (Table S1).

10.1128/mBio.01833-19.8 TABLE S1 Symbiotic phenotypes of *Lotus japonicus* wild type and mutant plants grown in Cologne soil (*n *= 5). Download Table S1, EPS file, 1.3 MB. Copyright © 2019 Thiergart et al. 2019 Thiergart et al. This content is distributed under the terms of the Creative Commons Attribution 4.0 International license.

In order to determine the impact of rhizobial and AM symbiosis on root microbiota assembly, we characterized fungal and bacterial communities of unplanted Cologne soil and the rhizosphere and root compartments of all above-mentioned *L. japonicus* genotypes at the bolting stage (∼10-week-old plants). Visible nodules and root primordia were removed from the roots of nodulating WT and *ram1* plants prior to sample processing for community profiling. We amplified the V5-V7 hypervariable region of the bacterial 16S rRNA gene and the ITS2 region of the eukaryotic ribosomal genes. High-throughput sequencing of these amplicons yielded 22,761,657 16S and 21,228,781 ITS reads, distributed in 222 and 274 samples, respectively, which were classified into 5,780 and 3,361 distinct microbial OTUs. Analysis of α-diversity revealed a general reduction of complexity from unplanted soil to the rhizosphere and finally to root compartments for bacterial communities, whereas the complexity of fungal communities was similar for the plant-associated compartments (Fig. S2A and B), which is consistent with a recent study of A. thaliana root-associated fungal communities (32). Bacterial α-diversity was slightly elevated for the *nfr5* genotype in rhizosphere and root compartments in comparison to all other genotypes (Fig. S2A). Fungal communities were similarly diverse in the rhizosphere of all tested plant genotypes, but their diversity in the root compartment was significantly and specifically reduced in all three AM mutants (*ccamk*, *ram1*, and *symrk*) (Fig. S2B).

10.1128/mBio.01833-19.2 FIG S2 Alpha and beta diversity across plant compartments and genotypes. (A) Shannon diversity indices for the bacterial (16S amplicon) data set. (B) Shannon diversity indices for the fungal (ITS2 amplicon) data set (*P* < 0.05 by ANOVA with Tukey’s *post hoc* test). (C) Principal-coordinate analysis of Bray-Curtis distances for the bacterial data set (*n* = 222). (D) Principal-coordinate analysis of Bray-Curtis distances for the fungal data set (*n* = 274). Download FIG S2, EPS file, 2.9 MB. Copyright © 2019 Thiergart et al. 2019 Thiergart et al. This content is distributed under the terms of the Creative Commons Attribution 4.0 International license.

**FIG 3:**
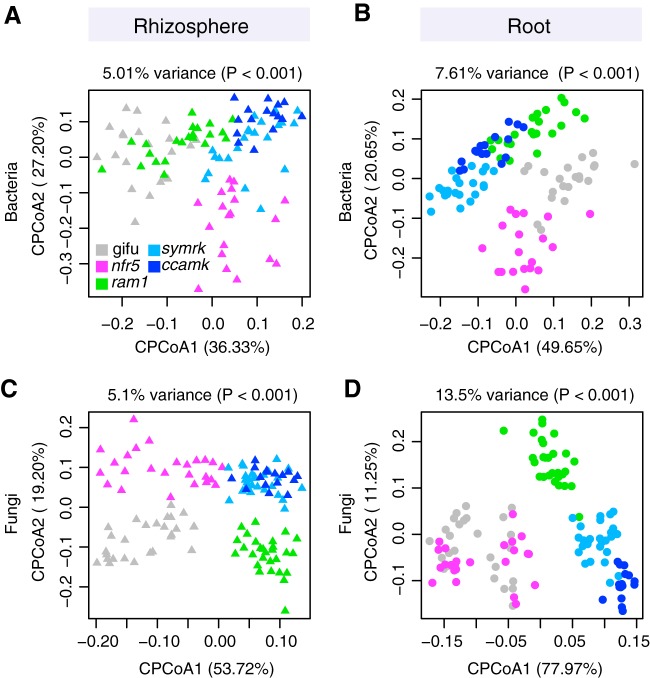
Constrained PCoA showing the effect of genotype on microbial communities. (A and B) Constrained PCoA plots for bacterial data sets showing rhizosphere samples (*n* = 100) (A) and root samples (*n* = 100) (B). (C and D) Constrained PCoA plots for fungal data sets showing only rhizosphere samples (*n* = 124) (C) and root samples (*n* = 122) (D) from *ram1* AMS-deficient, *nfr5* RNS-deficient, *symrk* AMS- and RNS-deficient, and *ccamk* AMS- and RNS-deficient plants.

Analysis of β-diversity using principal-coordinate analysis (PCoA) of Bray-Curtis distances showed a significant effect of soil batch on soil-resident bacterial and fungal communities (Fig. S2C and D). In order to account for this technical factor and assess the impacts of the different host compartment and genotypes on community composition, we performed a canonical analysis of principal coordinates (CAP) (36). This revealed a clear differentiation of bacterial and fungal communities of the tested plant genotypes in both root and rhizosphere compartments, with the host genotype explaining as much as 7.61% of the overall variance of the 16S rRNA and 13.5% of the ITS2 data (*P* < 0.001) (Fig. 3). The rhizosphere compartments of WT and *ram1* plants were found to harbor similar bacterial communities but were separate from those of *symrk* and *ccamk* plants (Fig. 3A). Furthermore, the rhizosphere communities of each of these four plant genotypes were found to be significantly different from that of *nfr5* plants (Fig. 3A). A similar trend was observed for fungal communities, except that WT and *ram1* rhizosphere communities were clearly separated from each other (Fig. 3C). For the root compartment, we found bacterial communities that were distinctive for each of the five plant genotypes (Fig. 3B). This genotype effect was also found in the root-associated fungal communities, with the exception of the *nfr5* community, which was indistinguishable from that of the WT (Fig. 3D). We then tested the contribution of AM and rhizobial symbionts to the observed patterns of diversity, in order to determine if AM fungi (Glomeromycota) and nitrogen-fixing Mesorhizobium loti (*Phyllobacteriaceae*) are the sole drivers of these host genotype community shifts (Fig. 3). We performed an *in silico* experiment in which sequencing reads of these two symbiotic taxonomic groups were removed from the analyses. Although we observed a decrease in the percentage of variance explained by host genotype (compare Fig. S3 to Fig. 3), overall patterns of β-diversity remained unaltered, suggesting that other community members besides root nodule and arbuscular mycorrhizal symbionts contribute to the plant genotype-specific community shifts. Collectively, our analyses of *L. japonicus* symbiotic mutants grown in natural soil show that lack of AMS and/or RNS has a significant effect on plant growth and on the structures of bacterial and fungal communities associated with legume roots.

10.1128/mBio.01833-19.3 FIG S3 CPCoA with *in silico* depletion of known symbionts. Results are separated by compartments. Data sets were constrained by genotype and filtered for effects of experiments and soil type. (A) Bacterial data set from which OTUs belonging to the *Phyllobacteriaceae* were removed before analysis (root, *n* = 100; rhizosphere, *n* = 100). (B) Fungal data set from which OTUs belonging to the Glomeromycota were removed before analysis (root, *n* = 122; rhizosphere, *n* = 124). Download FIG S3, EPS file, 1.4 MB. Copyright © 2019 Thiergart et al. 2019 Thiergart et al. This content is distributed under the terms of the Creative Commons Attribution 4.0 International license.

### Loss of symbiosis affects specific bacterial and fungal families of the root microbiota.

**FIG 4:**
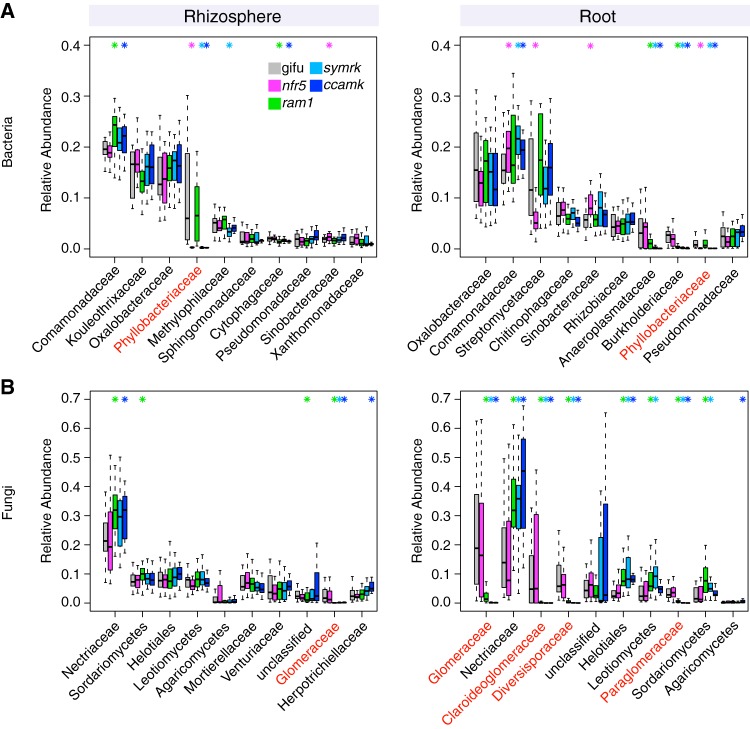
Relative abundances of the main microbial taxa across plant compartments and genotypes. (A) RAs for bacterial families in rhizosphere (left) and root (right) compartments. (B) RAs for fungal families in rhizosphere (left) and root (right) compartments. Taxa are sorted in decreasing order according to their average RA in WT plants (only the first 10 most abundant taxonomic groups are shown). RAs in the WT as well as the respective mutants are displayed. Significant differences compared to the WT are marked with an asterisk in the color of the mutant (*P* < 0.05 by a Kruskal-Wallis test). Families that include known symbionts are marked in red (*Phyllobacteriaceae* for bacteria and Glomeromycetes for fungi). For some fungal taxa, the next-highest rank is shown when no family-level information was available. Data for *ram1* AMS-deficient, *nfr5* RNS-deficient, *symrk* AMS- and RNS-deficient, and *ccamk* AMS- and RNS-deficient plants are shown.

Comparison of bacterial family abundances between the WT and mutants lacking RNS and/or AM symbiosis identified significant changes in members of the *Comamonadaceae*, *Phyllobacteriaceae*, *Methylophilaceae*, *Cytophagaceae*, and *Sinobacteraceae* in the rhizosphere compartment (top 10 most abundant families) (Fig. 4A). The abundance of *Comamonadaceae* and *Phyllobacteriaceae* also differed significantly in the root compartment of RNS mutants compared to the WT. *Streptomycetaceae* and *Sinobacteraceae* were specifically affected by the loss of *Nfr5*, whereas *Anaeroplasmataceae* and *Burkholderiaceae* were affected by the lack of AM symbiosis in *symrk* and *ccamk* plants (Fig. 4A). The relative abundances of the same two families were also significantly reduced in *ram1* roots, suggesting that active AM symbiosis influences root colonization by a subset of bacterial root microbiota taxa. Six out of the 10 most abundant fungal families in the rhizosphere compartment of *Lotus* plants belonged to the Ascomycota (Fig. 4B). In contrast, the root endosphere was dominated by numerous families of the Glomeromycota, which were found to be almost fully depleted from the rhizosphere and root compartments of *ram1*, *symrk*, and *ccamk* mutants, indicating that the absence of AM symbiosis predominantly affects Glomeromycota and does not limit root colonization by or rhizosphere association of other fungal families. However, the depletion of Glomeromycota in AM mutant roots was accompanied by an increase in the relative abundance of Ascomycota members belonging to the Nectriaceae in both rhizosphere and root compartments and by an increased abundance of unclassified Helotiales, Leotiomycetes, and Sordariomycetes members in the root compartment only (Fig. 4B).

**FIG 5:**
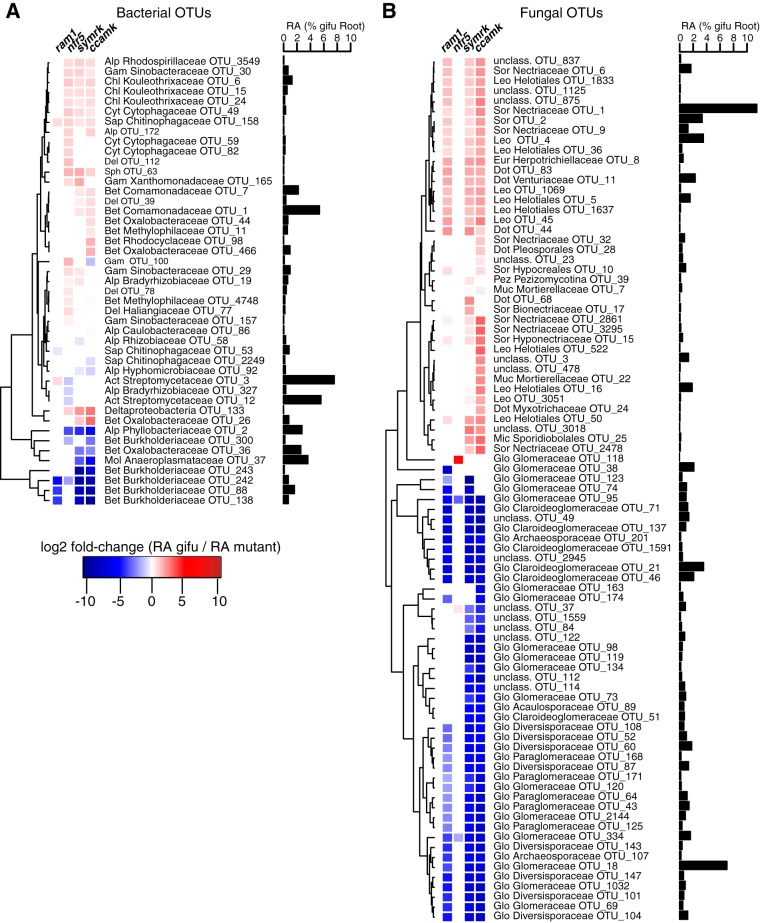
Differential abundance analysis of root-associated OTUs. (A) Dendrogram of bacterial OTUs that are differentially abundant in the roots of mutants compared to WT roots. (B) Dendrogram of fungal OTUs that are differentially abundant in the roots of mutants compared to WT roots. Only OTUs that have an average RA of >0.1% across all root samples, including mutants, are considered here. The dendrogram is based on hierarchical clustering. For each OTU, the fold change in RA from the WT to mutants is indicated (*P* < 0.05 by a Kruskal-Wallis test). Next to each OTU, the RA in WT roots is indicated. Phylum and family associations (if available) are given for each OTU. Abbreviations of bacterial phyla: Del, *Deltaproteobacteria*; Gem, Gemm-1; Chl, *Chloroflexi*; Bet, *Betaproteobacteria*; Alp, *Alphaproteobacteria*; Gam, *Gammaproteobacteria*; Cyt, *Cytophagia*; Sap, *Saprospiria*; Ped, *Pedosphaera*; Sph, *Sphingobacteria*; Mol, *Mollicutes*. Abbreviations of fungal phyla: Sor, Sordariomycetes; Dot, Dothideomycetes; Mic, Microbotryomycetes; Ust, Ustilaginomycetes; Eur, Eurotiomycetes; Leo, Leotiomycetes; Aga, Agaricomycetes; Glo, Glomeromycetes; Pez, Pezizomycotina; Muc, Mucoromycotina. Data for *ram1* AMS-deficient, *nfr5* RNS-deficient, *symrk* AMS- and RNS-deficient, and *ccamk* AMS- and RNS-deficient plants are shown.

Closer inspection of the microbial community shifts at the OTU level identified 45 bacterial OTUs and 87 fungal OTUs enriched in the roots of symbiosis mutants compared to those of the WT (Fig. 5) and 60 bacterial OTUs and 30 differentially abundant fungal OTUs in the rhizosphere samples (Fig. S4). The absence of RNS in *nfr5* roots affected the relative abundances of multiple OTUs (*n* = 27 in the root; *n* = 23 in the rhizosphere) belonging to diverse taxa. Many of these OTUs (*n* = 18 in the root; *n* = 16 in the rhizosphere) showed similar differential relative abundances in *symrk* and/or *ccamk* mutants compared to the WT (Fig. 5A), indicating that their contribution to the *Lotus* root communities outside nodules is affected by active nitrogen-fixing symbiosis. Impairment of both AMS and RNS in *symrk* and/or *ccamk* mutants resulted in opposite changes in the relative root abundances of OTUs belonging to specific *Burkholderiales* families. The depletion of OTUs belonging to the *Burkholderiaceae* (*n* = 5) was accompanied by an enrichment of OTUs from other *Burkholderiales* families (*Oxalobacteraceae* [*n* = 3], *Comamonadaceae* [*n* = 2], and *Methylophilaceae* [*n* = 2]) (Fig. 5A). Only three of the above-mentioned *Burkholderiaceae* OTUs were depleted in *ram1* roots, suggesting that their enrichment in *Lotus* roots is dependent on functional AM symbiosis.

10.1128/mBio.01833-19.4 FIG S4 Differential abundance analysis of rhizosphere-associated OTUs showing enrichment and depletion in mutants. (A) Dendrogram of bacterial OTUs that are differentially abundant in the rhizosphere of mutants compared to the WT. (B) Dendrogram of fungal OTUs that are differentially abundant in the rhizosphere of mutants compared to the WT. Only OTUs that have an average RA of >0.1% across all rhizosphere samples, including mutants, are considered here. The dendrogram is based on hierarchical clustering. For each OTU, the fold change in the RA from the WT to mutants is indicated (*P* < 0.05 by a Kruskal-Wallis test). Next to each OTU, the RA in the WT rhizosphere is indicated. Phylum and family associations are given for each OTU. Abbreviations of bacterial phyla: Del, *Deltaproteobacteria*; Gem, Gemm-1; Chl, *Chloroflexi*; Bet, *Betaproteobacteria*; Alp, *Alphaproteobacteria*; Gam, *Gammaproteobacteria*; Cyt, *Cytophagia*; Sap, *Saprospiria*; Ped, *Pedosphaera*; Sph, *Sphingobacteria*; Mol, *Mollicutes*. Abbreviations of fungal phyla: Sor, Sordariomycetes; Dot, Dothideomycetes; Mic, Microbotryomycetes; Ust, Ustilaginomycetes; Eur, Eurotiomycetes; Leo, Leotiomycetes; Aga, Agaricomycetes; Glo, Glomeromycetes; Pez, Pezizomycotina; Muc, Mucoromycotina. Download FIG S4, EPS file, 2.4 MB. Copyright © 2019 Thiergart et al. 2019 Thiergart et al. This content is distributed under the terms of the Creative Commons Attribution 4.0 International license.

Analysis of the ITS2 amplicon sequences from root samples identified a large number of Glomeromycota OTUs (*n* = 39), demonstrating the capacity of *Lotus* Gifu roots grown in natural soil to accommodate a phylogenetically diverse community of AM fungi (Fig. 5B). The majority of these fungal OTUs (*n* = 31) were depleted in *symrk*, *ccamk*, and *ram1* mutant roots, indicating that their enrichment is dependent on a functional AM symbiosis pathway. Their intraradical colonization appears to be independent of *RAM1*, as 12 OTUs that were assigned to the Glomeromycota or unclassified, 9 of which define a Glomeromycota sublineage, were depleted in *symrk* and *ccamk* but not in *ram1* roots. The reduced abundance of Glomeromycota OTUs in the endosphere compartment was accompanied by an increased abundance of Ascomycota members, especially of members belonging to the Nectriaceae (8 OTUs) and Helotiales (7 OTUs) families, which is suggestive of a mutually exclusive occupancy of the intraradical niche. In sum, our results reveal that for *Lotus* plants grown in natural soil, CSSP genes are essential for root colonization by a wide range of Glomeromycota fungi and that these genes significantly affect the abundances of multiple bacterial taxa, predominantly belonging to the *Burkholderiales* and *Rhizobiales* orders.

In order to assess the impact of mutations of *Lotus* symbiotic genes on microbial interactions, we constructed cooccurrence microbial networks for each genotype independently using SparCC (37) (Fig. S5). We observed an increase in the number of edges of the networks inferred from *symrk* and *ccamk* networks (748 and 805 edges, respectively) compared to Gifu WT, *nfr5*, and *ram1* networks (471, 569, and 500 edges, respectively) (Fig. S5A), despite comparable numbers of nodes for all genotypes. This unexpected observation suggests a greater connectivity between bacterial root commensals when both fungal and bacterial symbioses are disrupted in *symrk* and *ccamk* roots. In the corresponding five fungal networks, the number of OTUs is moderately reduced in *ram1* and approximately halved in *symrk* and *ccamk* networks (86 in Gifu WT, 78 in *nfr5*, 63 in *ram1*, 39 in *symrk*, and 41 in *ccamk* networks) (Fig. S5A), which can be explained by the partial or complete depletion of Glomeromycota taxa in the latter three host genotypes. This decrease in the number of fungal OTUs is accompanied by a decrease in the number of edges in the fungal networks (329 edges for Gifu, 363 for *nfr5*, 231 for *ram1*, 101 for *symrk*, and 117 for *ccamk*) (Fig. S5A). To directly compare the numbers of edges between plant genotypes for bacterial and fungal networks, we first normalized the number of bacterial and fungal OTUs (Fig. S5B). Compared to Gifu WT and *nfr5* networks, the degree centrality for bacterial OTUs is slightly increased in the *ram1* network (significant only for positive correlations) and clearly increased in *symrk* and *ccamk* networks (significant for both positive and negative correlations), supporting the above-mentioned change in the network structure of the bacterial root microbiota when both fungal and bacterial symbioses are disrupted in *Lotus* roots. In contrast, the degree centrality of fungal OTUs remains mostly stable across fungal networks identified in the five plant genotypes. Together, our analyses suggest that the combined activities of fungal and bacterial symbioses negatively influence the connectivity within the *Lotus* bacterial root microbiota.

10.1128/mBio.01833-19.5 FIG S5 Network analysis of root-associated bacterial and fungal OTUs. (A) SparCC-based networks for WT and mutant roots (top, bacterial OTUs; bottom, fungal OTUs). Nodes are colored by phylum and are ordered by increasing degree (clockwise, degree-sorted circle layout from Cytoscape). Positive connections are in blue, and negative connections are in red. All connections are significant (pseudo-*P* values of <0.05 by SparCC). (B) Box plots showing degree centrality for the different networks (left, bacteria; right, fungi). Degree centrality was calculated separately for positive and negative edges, and statistical tests were done separately (false discovery rate [FDR] of <0.05 by a pairwise Wilcox test). Download FIG S5, EPS file, 1.9 MB. Copyright © 2019 Thiergart et al. 2019 Thiergart et al. This content is distributed under the terms of the Creative Commons Attribution 4.0 International license.

## DISCUSSION

Here, we investigated the role of host AMS and/or RNS genes in establishing structured bacterial and fungal communities in the rhizosphere and endosphere compartments of *L. japonicus* grown in natural soil. Impairment of RNS in *nfr5* or AMS in *ram1* plants had a significant impact on root microbiota structure, which was mainly, but not exclusively, confined to the composition of the corresponding bacterial or fungal communities, respectively (Fig. 3 to 5).

The shift between the root-associated microbial communities of the WT and the *nfr5-2* mutant is in line with both the qualitative and quantitative findings of a previous report on the *Lotus* bacterial root microbiota (Fig. 3A and B) (22). Here, however, we observed a more distinctive rhizosphere community in both WT and *nfr5* plants, also leading to a less prominent community shift in this compartment (see Fig. S6 in the supplemental material), which was not previously observed. These differences in rhizosphere bacterial composition are likely caused by a soil batch effect and, to a lesser extent, possibly also the use of different sequencing platforms (Illumina in this study versus 454 pyrosequencing in reference 22). The nearly unaltered fungal community composition in *nfr5* mutant plants compared to the WT (only 3 out of 39 Glomeromycota OTUs were differentially abundant) suggests that NFR5 is dispensable for fungal colonization of *L. japonicus* roots. This is consistent with recent findings from analyses of diverse AM symbiotic mutants of *Lotus* where the structures of the root-associated fungal communities of AM- and CSSP-deficient mutants were indistinguishable (25). Despite unaltered fungal communities in *nfr5* mutants, we found a marked shoot biomass reduction for this genotype grown in natural soil (∼4-fold) (Fig. 2), revealing that intraradical colonization by soil-derived fungal endophytes is robust against major differences in plant growth.

10.1128/mBio.01833-19.6 FIG S6 Ternary plots showing compartment-enriched bacterial OTUs. Plots are shown separately for WT and mutant plants. Below each plot, the number of enriched OTUs for each compartment is indicated. Download FIG S6, EPS file, 2.5 MB. Copyright © 2019 Thiergart et al. 2019 Thiergart et al. This content is distributed under the terms of the Creative Commons Attribution 4.0 International license.

A recent microbial multikingdom interaction study in *A. thaliana* showed that bacterial commensals of the root microbiota are crucial for the growth of a taxonomically wide range of fungal root endophytes. These antagonistic interactions between bacterial and fungal root endophytes are essential for plant survival in natural soil (32). We have shown here that the almost complete depletion of diverse Glomeromycota taxa from roots of each of the three AM mutants was accompanied by an enrichment of fungal OTUs belonging to the families Nectriaceae and Helotiales (Fig. 4). We speculate that the increased relative abundance of these fungal taxa is caused by intraradical niche replacement as a compensatory effect following the exclusion of Glomeromycota symbionts from the root compartment. Previous monoassociation experiments have shown that isolates belonging to the Nectriaceae and Helotiales can have either mutualistic or pathogenic phenotypes (38–40). Given that all plant genotypes were free of disease symptoms when grown in natural soil (Fig. 2), we speculate that the complex shifts in the compositions of the bacterial root microbiota in *nfr5*, *symrk*, and *ccamk* mutants did not affect the capacity of bacterial endophytes to prevent pathogenic fungal overgrowth. Of note, Helotiales root endophytes were also enriched in roots of healthy Arabis alpina, a nonmycorrhizal plant species and relative of *A. thaliana*, and contributed to phosphorus nutrition of the host when grown in extremely phosphorus-impoverished soil (41). The enrichment of Helotiales in *Lotus* AM mutants is therefore consistent with potential niche replacement by other fungal lineages to ensure plant nutrition in nutrient-impoverished soils. Although the proposed compensatory effect in AM mutants will need further experimental testing in phosphorus-depleted soils, our hypothesis is consistent with the only mild impairment of plant growth in *ram1* mutants (Fig. 2).

We observed that members of the bacterial families *Burkholderiaceae* and *Anaeroplasmataceae* are significantly depleted in the roots of each of the three AM mutants compared to the WT. Members of the Glomeromycota have been found to contain intracellular endosymbiotic bacteria (42), with some belonging to the order *Burkholderiales* (56). Interestingly, the most positively correlated bacterial OTUs with Glomeromycota fungi in our network analyses included one *Anaeroplasmataceae* and two *Burkholderiaceae* OTUs (Fig. S7), further indicating a direct interaction between these taxonomic groups. These findings suggest either that these bacteria are endosymbionts of Glomeromycota fungi that are excluded from the roots of the AM-defective genotypes or that their intraradical colonization is indirectly mediated by AM fungus infection. Except for small changes in the bacterial root microbiota in *ram1* plants, which are mainly limited to the above-mentioned *Burkholderiaceae* and *Anaeroplasmataceae* OTUs, the structure of the root-associated bacterial community is remarkably robust against major changes in the composition of root-associated fungal assemblages (Fig. 5). Nevertheless, we observed clear increases in connectivity between bacterial OTUs and degree centrality parameters in the bacterial networks constructed from *symrk* and *ccamk* mutants compared to those of Gifu, *nfr5*, and *ram1* plants. This unexpected change in bacterial network structure could be a consequence of a vacant niche created by the depletion of dominant Glomeromycota taxa from the interior of *symrk* and *ccamk* roots. But niche filling by bacterial commensals is unlikely to explain the observed alteration in bacterial network connectivity because Glomeromycota root colonization is greatly diminished in *ram1* plants, without major changes in the corresponding bacterial network structure (Fig. 4 and Fig. S5). The increased bacterial network connectivity in *symrk* and *ccamk* roots is more likely a consequence of the inactivation of the CSSP, which remains intact in all other tested genotypes. However, we cannot fully exclude that the altered nutritional status in *symrk* and *ccamk* plants resulting from the combined loss of host and symbiont metabolic activities of and induced by both symbionts also plays a role in the altered network structure.

10.1128/mBio.01833-19.7 FIG S7 Interkingdom correlation of bacterial OTUs in wild-type roots. Bacterial OTUs that show a significant correlation with fungal OTUs across wild-type root samples (OTU RA of >0.01%; Spearman rank correlation *P* value of <0.001) are sorted according to their cumulative correlation with all fungal OTUs (gray bars). In addition, the cumulative correlation with only Glomeromycota OTUs is indicated by blue dots within each bar. Taxonomy at the family level is given for each OTU, if available. OTUs belonging to the *Burkholderiaceae* and *Anaeroplasmataceae* are highlighted in red. Download FIG S7, EPS file, 1.0 MB. Copyright © 2019 Thiergart et al. 2019 Thiergart et al. This content is distributed under the terms of the Creative Commons Attribution 4.0 International license.

Paleontological and phylogenomic studies established the ancestral origin of genetic signatures enabling AM symbiosis in land plants (1, 43). In monocots and dicots, the extended AM fungal network is primarily recognized as a provider of nutrients, particularly phosphorus (44, 45), but the positive impact of AM symbiosis on the host transcends nutrient acquisition (46). Additionally, phylogenomic studies of the symbiotic phosphate transporter PT4 suggest that this trait evolved late and therefore that phosphorus acquisition might not have been the (only) driving force for the emergence of AM symbiosis (43). *SymRK* and *Ram1* were identified in the genomes of liverworts, but the evolution of *CCaMK* predated the emergence of all land plants, as shown by its presence and conserved biochemical function in advanced charophytes (43). Together, these findings raise questions regarding the forces driving the evolution of signaling genes enabling intracellular symbioses in land plants. Our study shows that in *L. japonicus*, the simultaneous impairment of AM and RN symbioses in *symrk* and *ccamk* plants had a dramatic effect on the composition of both bacterial and fungal communities of the legume root microbiota (Fig. 5). Importantly, mutation of *CCaMK* and *SymRK* led to an almost complete depletion of a large number of fungal OTUs, mostly belonging to the Glomeromycota, indicating that in *Lotus*, these genes predominantly control the colonization of roots by this particular fungal lineage. The finding that *ram1-2* mutants show retained accommodation for a subset of fungal root endophytes (*n* = 13) (Fig. 4B and Fig. 5B) whose colonization is dependent on an intact common symbiosis pathway is not surprising based on the capacity of these mutants to enable fungal colonization but not to sustain a full symbiotic association (35) and indicates that *RAM1* is dispensable for intraradical colonization by these Glomeromycota fungi. Alternatively, these fungal root endophytes may engage in commensal rather than mutualistic relationships with *L. japonicus* independently of the AM symbiosis pathway, as is the case for multiple species of commensal nonsymbiotic rhizobia (22, 47). Given that *ram1* mutants specifically block AM arbuscule differentiation but not root colonization (35), it is conceivable that the Glomeromycota taxa colonizing this plant genotype cannot form arbuscules during root colonization.

Legumes have evolved the capacity to recognize and accommodate both types of intracellular symbionts, and the large effect of CSSP genes on associated microbiota seen in the present work could reflect a legume-specific trait. However, in rice, which does not engage in symbiotic relationships with nodulating rhizobia, mutants lacking *CCaMK* were also found to display significant changes in root-associated bacterial communities that could be mainly explained by the depletion of *Rhizobiales* and *Sphingomonadales* lineages (48). Thus, our findings based on comparative microbiota analysis of *Lotus ccamk* and *ram1* mutants suggest a broader role for common symbiosis signaling genes in microbiota assembly. Future studies on orthologous genes in basal land plants will contribute to a better understanding of the role of symbiotic signaling in the evolution of plant-microbiota associations.

## MATERIALS AND METHODS

### Preparation and storage of soil.

The two soil batches used in this study were collected from the Max Planck Institute for Plant Breeding Research agricultural field located in Cologne, Germany (50.958N, 6.865E), in the following seasons: spring/autumn 2016 for CAS11 soil and spring 2017 for CAS12 soil (CAS indicates Cologne agriculture soil). The field had not been cultivated in previous years, and no fertilizer or pesticide administration took place at the harvest site. Following harvest, soil was sieved, homogenized, and stored at 4°C for further use.

### Soil and plant material.

All studied *L. japonicus* symbiosis-deficient mutants, *nfr5-2* (33), *ram1-2* (35), *symrk-3* (7), and *ccamk-13* (34), originated from the Gifu B-129 genotype.

### Plant growth and harvesting procedure.

The germination procedure for *L. japonicus* seeds included sandpaper scarification and surface sterilization in 1% hypochlorite bleach (20 min at 60 rpm), followed by three washes with sterile water and incubation on wet filter paper in petri dishes for 1 week (temperature of 20°C, day/night cycle of 16/8 h, and relative humidity of 60%). For each genotype and soil batch, six to eight biological replicates were prepared by potting four plants in a 7- by 7- by 9-cm pot filled with the corresponding batch of soil (six replicates for CAS11 soil and eight replicates for CAS12 soil). For each batch of soil, two independent experiments were carried out. Plants were incubated for 10 weeks (until the bolting stage) in a greenhouse (day/night cycle of 16/8 h, light intensity of 6,000 lx, temperature of 20°C, and relative humidity of 60%) and watered with tap water twice per week.

The block of soil containing plant roots was removed from the pot, and adhering soil was discarded manually. Three sample pools were collected: complete root systems (harvested 1 cm below the hypocotyl), upper fragments of the root systems (4 cm-long, starting 1 cm below the hypocotyl), and lower root system fragments (harvested from 9 cm below the hypocotyl) (the latter two were collected from plants grown in the same pot) (Fig. 1A). All pools were washed twice with sterile water containing 0.02% Triton X-100 detergent and twice with pure sterile water by vigorous shaking for 1 min. The rhizosphere compartment was derived by collection of the pellet following centrifugation of the first wash solution for 10 min at 1,500 × *g*. The nodules and visible primordia were separated from washed root pools of nodulating genotypes (WT and *ram1-2*) with a scalpel and discarded. In order to obtain the root compartment, the root sample pools were sonicated to deplete the microbiota fraction attached to the root surface. This included 10 cycles of 30-s ultrasound treatment (Bioruptor NextGen UCD-300; Diagenode) for complete root systems and upper root fragments, while for the lower root fragments, the number of cycles was reduced to 3. All samples were stored at −80°C for further processing. For AM colonization inspection, the whole root system of washed soil-grown plants was stained with 5% ink in a 5% acetic acid solution and inspected for intraradical infection.

### Generation of 16S rRNA and ITS2 fragment amplicon libraries for Illumina MiSeq sequencing.

Root pool samples were homogenized by grinding in a mortar filled with liquid nitrogen and treatment with a Precellys24 tissue lyser (Bertin Technologies) for two cycles at 5,600 rpm for 30 s. DNA was extracted with the FastDNA spin kit for soil, according to the manufacturer’s protocol (MP Bioproducts). DNA concentrations were measured fluorometrically (Quant-iT PicoGreen double-stranded DNA [dsDNA] assay kit; Life Technologies, Darmstadt, Germany) and adjusted to 3.5 ng/μl. Barcoded primers targeting the variable V5-V7 region of the bacterial 16S rRNA gene (799F and 1193R [29]) or targeting the ITS2 region of the eukaryotic ribosome (fITS7 and ITS4 [30, 31]) were used for amplification. The amplification products were purified, pooled, and subjected to sequencing with Illumina MiSeq equipment.

### Processing of 16S rRNA and ITS2 reads.

Libraries from the three root fractions (including the root tip endosphere, the upper root endosphere, and the whole-root endosphere) were analyzed independently. Due to a very low read count for 16S data in the first experiment in CAS11 soil, these data were not included in the final analysis. This resulted in an overall lower sample number for bacteria than for fungi (222 versus 274 samples). All sets of amplicon reads were processed as recently described (32), using a combination of QIIME (49) and USEARCH (50) tools. For both data sets, paired-end reads were used. For ITS2 data, forward reads were kept, in case no paired version was available. Main steps include quality filtering of reads, dereplication, chimera detection, and OTU clustering at a 97% threshold. 16S reads were filtered against the Greengenes database (51), whereas for ITS2, the reads were checked with ITSx (52) and compared against a dedicated ITS database to remove ITS sequences from nonfungal species. Taxonomic classification was done with uclust (assign_taxonomy from QIIME) for 16S OTUs and rdp classifier (53) for ITS2 OTUs. For the sake of consistency with NCBI taxonomic classification, the assignment of the ITS2 sequences was manually corrected so that that all OTUs assigned as *Ilyonectria* were assigned as belonging to the Sordariomycetes, Hypocreales, and Nectriaceae. For 16S data, OTUs assigned as mitochondrial or chloroplast were removed prior to analysis.

### Statistical analysis.

For calculating Shannon diversity indices, OTU tables were rarefied to 1,000 reads (single_rarefaction.py from QIIME; samples with fewer than 1,000 reads were omitted). Significant differences were determined using analysis of variance (ANOVA) (aov function in R) and a Tukey *post hoc* test (Tukey honestly significant difference [HSD] test in R; *P* < 0.05). For calculating Bray-Curtis distances between samples, OTU tables were normalized using cumulative sum scaling (CSS) (54). Bray-Curtis distances were used as the input for principal-coordinate analysis (PCoA) (cmdscale function in R) plots and as the input for constrained analysis of principal coordinates (CPCoA) (capscale function, vegan package in R). For the latter, the analysis was constrained by genotypes (each mutant and the WT separately) and corrected for the effect of the two soil types (CAS11 and CAS12) and the four individual experiments (using the “Condition” function). This analysis was repeated with OTU tables from which OTUs that represent known plant symbionts (*Phyllobacteriaceae* for 16S and Glomeromycota for ITS2) were removed before normalization, distance calculation, and CPCoA. A previously described approach was used to draw ternary plots and for respective enrichment analysis (22). The fold change of OTUs between WT and mutant plants was calculated as follows. Samples showing a read count of <5,000 were removed. OTUs with a mean relative abundance (RA) of >0.1% across all root or rhizosphere samples were kept for analysis. The fold change in RA from the WT to mutants was calculated over all WT samples for *nfr5*, *ram1*, and *symrk*, whereas the change for *ccamk* was calculated only with WT samples from experiments where *ccamk* mutants were present. To avoid zeros in the calculation, the RA of OTUs missing from samples was set to 0.001%. The significance of differences in abundance was tested using the Kruskal-Wallis test (*P* < 0.05). Networks for each genotype and kingdom were calculated independently using SparCC (37). OTU tables were filtered before analysis to include only samples from one soil type (CAS12) to avoid biases. In addition, only OTUs that were present in more than 10 samples and had a mean RA of >0.1% were kept for network analysis. Raw count tables were given to SparCC as an input, and the resulting correlations were filtered by significance (*P* < 0.05). Networks were drawn using Cytoscape (55). To calculate the degree centrality, the number of positive and negative connections for each OTU was divided by the number of OTUs present in the respective network. Correlations between bacterial and fungal OTUs were calculated as follows. OTUs that appeared in fewer than 10 Gifu root samples and had a mean RA of < 0.1% were not considered for this analysis. Spearman rank correlations were calculated between RA values of bacterial and fungal OTUs across all Gifu root samples (cor.test function in R; *P* < 0.001). To show the cumulative correlation of bacterial OTUs with fungal OTUs, the respective correlations for one bacterial OTU were summed so that the number of correlations and the strength could be assessed in one analysis. This was repeated but just for fungal OTUs annotated as belonging to the Glomeromycota.

### Data availability.

All sequencing data are available at the European Nucleotide Archive (ENA). Bacterial reads are accessible under project accession no. PRJEB34100, and fungal reads are available under project accession no. PRJEB34099. Relevant data files (e.g., OTU tables) can be found at GitHub (https://github.com/ththi/Lotus-Symbiosis).
